# Histidine-rich protein 2 (*pfhrp2*) and *pfhrp3* gene deletions in *Plasmodium falciparum* isolates from select sites in Brazil and Bolivia

**DOI:** 10.1371/journal.pone.0171150

**Published:** 2017-03-16

**Authors:** Giselle Maria Rachid Viana, Sheila Akinyi Okoth, Luciana Silva-Flannery, Danielle Regina Lima Barbosa, Alexandre Macedo de Oliveira, Ira F. Goldman, Lindsay C. Morton, Curtis Huber, Arletta Anez, Ricardo Luiz Dantas Machado, Luís Marcelo Aranha Camargo, Suiane Costa Negreiros do Valle, Marinete Marins Póvoa, Venkatachalam Udhayakumar, John W. Barnwell

**Affiliations:** 1 Instituto Evandro Chagas – IEC/SVS/MS, Ananindeua, Pará, Brasil; 2 Malaria Branch, Division of Parasitic Diseases and Malaria, Center for Global Health, Centers for Disease Control and Prevention, Atlanta, Georgia, United States of America; 3 Atlanta Research and Education Foundation, Decatur, Georgia, United States of America; 4 Pan American Health Organization, La Paz, Bolivia; 5 ICB5/USP e Faculdade São Lucas, Porto Velho, Rondônia, Brasil; 6 Secretaria Estadual de Saúde do Acre, Hemonúcleo Cruzeiro do Sul, Cruzeiro do Sul, Acre, Brasil; Quensland University of Technology, AUSTRALIA

## Abstract

More than 80% of available malaria rapid diagnostic tests (RDTs) are based on the detection of histidine-rich protein-2 (PfHRP2) for diagnosis of *Plasmodium falciparum* malaria. Recent studies have shown the genes that code for this protein and its paralog, histidine-rich protein-3 (PfHRP3), are absent in parasites from the Peruvian Amazon Basin. Lack of PfHRP2 protein through deletion of the *pfhrp2* gene leads to false-negative RDT results for *P*. *falciparum*. We have evaluated the extent of *pfhrp2* and *pfhrp3* gene deletions in a convenience sample of 198 isolates from six sites in three states across the Brazilian Amazon Basin (Acre, Rondonia and Para) and 25 isolates from two sites in Bolivia collected at different times between 2010 and 2012. *Pfhrp2* and *pfhrp3* gene and their flanking genes on chromosomes 7 and 13, respectively, were amplified from 198 blood specimens collected in Brazil. In Brazil, the isolates collected in Acre state, located in the western part of the Brazilian Amazon, had the highest percentage of deletions for *pfhrp2* 25 (31.2%) of 79, while among those collected in Rondonia, the prevalence of *pfhrp2* gene deletion was only 3.3% (2 out of 60 patients). In isolates from Para state, all parasites were *pfhrp2*-positive. In contrast, we detected high proportions of isolates from all 3 states that were *pfhrp3*-negative ranging from 18.3% (11 out of 60 samples) to 50.9% (30 out of 59 samples). In Bolivia, only one of 25 samples (4%) tested had deleted *pfhrp2* gene, while 68% (17 out of 25 samples) were *pfhrp3*-negative. Among the isolates tested, *P*. *falciparum pfhrp2* gene deletions were present mainly in those from Acre State in the Brazilian Amazon. These results indicate it is important to reconsider the use of PfHRP2-based RDTs in the western region of the Brazilian Amazon and to implement appropriate surveillance systems to monitor *pfhrp2* gene deletions in this and other parts of the Amazon region.

## Introduction

The number of reported malaria cases in Brazil has steadily decreased from 637,000 in 1999 to approximately 178,546 in 2013 [1, 2]. Most of the malaria cases in Brazil are caused by *Plasmodium vivax* (82%), are transmitted by *Anopheles darlingi* and occur in the Amazon region [1, 3]. The percentage of malaria infections due to *P*. *falciparum* has also declined somewhat in the past few years, from 21.5% of reported cases in 2009 to 18% in 2013 [3]. Bolivia, which borders Brazil, is similar in that the majority of malaria cases are due to *P*. *vivax* and only 7% are due to *P*. *falciparum* infection. Only a total of 7,342 malaria cases were reported in Bolivia in 2013 [4].

Microscopic examination of thick blood films remains the primary diagnostic method for malaria in Brazil and Bolivia, with numerous laboratories located throughout the Amazon region to support microscopic diagnosis. Nevertheless, malaria rapid diagnostic tests (RDTs) serve as an important component of primary malaria diagnosis in remote regions of the Amazon rainforest where it is challenging to establish and maintain laboratories staffed with well-trained microscopists. Malaria RDTs are individual test kits that can detect *Plasmodium*-specific antigens in a small volume (5 to 10 μL) of fresh blood using lateral flow immunochromatography [5]. RDTs offer a feasible alternative to microscopy as they do not require a laboratory or special equipment, are simple to use, and provide a positive or negative result within 20 minutes [5]. Indeed, the National Malaria Control Program (NMCP) in Brazil recommends the use of RDTs in these remote conditions [6]. The sensitivity or level of detection for malaria RDTs used to be 95% or even greater for *P*. *falciparum* densities of 100/μL, as the World Health Organization (WHO) recommended [7]. Malaria RDTs are designed to detect one or more abundant parasite antigens using specific monoclonal antibodies. Monoclonal antibodies to histidine-rich protein 2 (PfHRP-2) and to a specific *P*. *falciparum* epitope in parasite lactate dehydrogenase (Pf-pLDH) are specific for *P*. *falciparum* parasites. Monoclonal antibodies to a *P*. *vivax*-specific epitope (Pv-pLDH) are specific for detecting *P*. *vivax*, and antibodies to conserved epitopes in pLDH and aldolase commonly recognize all human malaria-causing *Plasmodium* species [8]. Currently there are over 100 commercially available malaria RDT brands produced by several dozen manufacturers. Malaria RDTs are categorized as two-, three- or four-band products, depending on the number of lines (“bands”) that may become visible on the strip, with one band always representing the control line. The most commonly used RDT by the Brazilian Ministry of Health is the SD Bioline Ag Pf/Pan 05FK60 test (Standard Diagnostics Inc., Kyonggi-do, Korea), which is a three-band RDT that targets PfHRP2 and pan-pLDH [9].

PfHRP2 is an abundant protein produced in all blood stages of *P*. *falciparum* [10], and is notable for a number of alanine- and histidine-rich repeats [11]. The protein is encoded by the *pfhrp2* gene that is 1063 bp long (based on 3D7 strain sequence) and located in the subtelomeric region of chromosome 8 [11–13]. It is immediately flanked by a *Plasmodium* exported protein (PHIST) of unknown function (PlasmoDB ID: PF3D7_0831900) and a putative heat shock protein 70 gene, PF3D7_0831700. A structural paralog, *pfhrp3*, is 977 bp (based on 3D7 sequence), located subtelomerically on chromosome 13 and is flanked on its 5’ end by a gene coding for a *Plasmodium* exported protein (PHISTb) of unknown function, PF3D7_1372100. A gene coding for acyl-CoA synthetase (PF3D7_1372400) is located approximately 9.1 kb from the 3’ end of *pfhrp3*. In certain instances, PfHRP2-based RDTs may cross-react with PfHRP3 because the latter protein can share sequence and epitope similarity with PfHRP2 [14, 15].

Recent reports from Peru [16, 17] have shown that *P*. *falciparum* isolates with *pfhrp2* and/or *pfhrp3* genes deletions are present in the Peruvian Amazon. Analysis of *pfhrp*2 and *pfhrp3* genes in isolates from French Guiana collected from 2010 to 2011 showed only 7.4% of isolates with deletions for *pfhrp3* and none for *pfhrp2* gene [18]. In Central America, one study conducted in Puerto Lempira province in Honduras determined that approximately 50.0% of the specimens tested had deleted *pfhrp3* gene but not *pfhrp2* [19]. Studies from countries outside the Americas such as Mali [20], Senegal [21] and India [22] have suggested that *P*. *falciparum* isolates circulating in those regions may also be *pfhrp2* negative, albeit only rarely or in much lower proportions. Deletion of the *pfhrp2* gene has led to false-negative RDT results when RDTs based exclusively on detecting PfHRP2 are used [5]. A recent case of *P*. *falciparum* malaria in a French citizen who had traveled to the Brazilian Amazon region was initially misdiagnosed as non-falciparum malaria after a combo RDT did only elicited a positive band for the *Plasmodium* aldolase antigen and not for the PfHRP2 [23]. *P*. *falciparum* infection was later confirmed after expert microscopic examination of blood smears and a pan-specific and *P*. *falciparum*-specific pLDH-based combination RDT was used [23]. This isolate was proven to be *pfhrp2* gene negative [23].

Investigation of the extent of *pfhrp2* and *pfhrp3* gene deletions in the Amazon River basin of Brazil and Bolivia was deemed a priority, particularly, since the prevalence of *pfhrp2*-negative isolates in the bordering Peruvian Amazon region has been reported to be as high as 40% [17]. This study provides an opportunity to evaluate occurrence of *pfhrp2* and *pfhrp3* genes, along with their respective flanking genes deletion in three different regions across the Brazilian Amazon Basin and in a province located in the eastern part of neighboring Bolivia. The genetic information that was obtained for Brazilian isolates was also compared to the results of serological studies that determined the presence and levels of the PfHRP2 in the corresponding patient plasma. It was anticipated that the information from this study would be useful for guiding the National Malaria Control Programs in the implementation of appropriate malaria RDTs in Brazil and Bolivia. Establishing the geographical distribution of *pfhrp2-* and *pfhrp3-* deleted parasites may help determine in what regions the use of PfHRP2-based RDTs should be reconsidered.

## Materials and methods

### Collection sites

This evaluation took place in three geographical areas in Brazil (Acre, Rondonia, and Para States in Brazil) and one in Bolivia (Beni Department). We selected areas to provide information from different malarious regions of these two countries. We aimed to collect 100 samples in each geographical area; this allowed us to detect a prevalence of gene deletion of 40% with 10% precision and confidence level of 95%. To facilitate sample collection, we chose to include three health facilities in Acre, two in Para, a single site in Rondonia and finally two facilities in Beni Department in Bolivia. We selected health facilities among the ones with the highest number of malaria cases in each area. Since those health facilities in each area were in close proximity (<100 Km from each other), we considered them together for the analysis of each geographical area without adjustment for sampling strategy or clustering within a geographical area.

**Fig 1:**
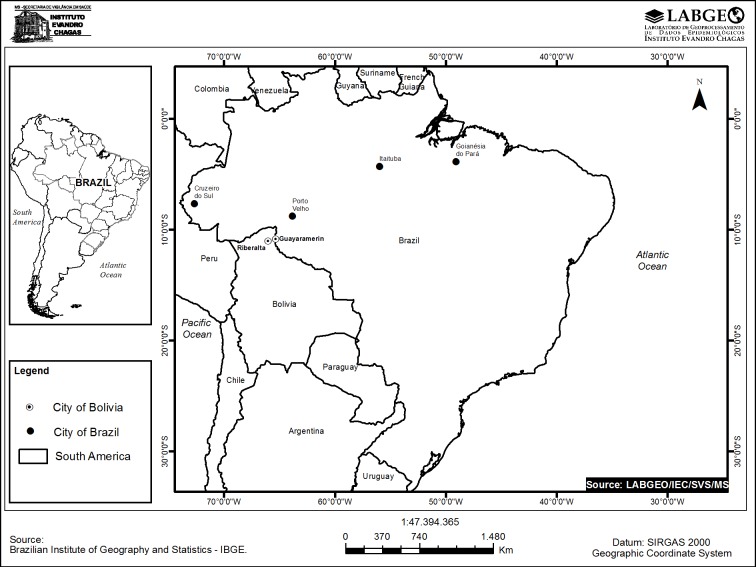
Study sites. Locations of study sites from Brazil and Bolivia are indicated.

Sample collection in Acre State, located on the western edge of the Amazon region of Brazil and bordering Peru, was carried out in 2012 at three health posts in the municipality of Cruzeiro do Sul. Samples from Rondonia State, located in the southwest portion of the Brazilian Amazon basin bordering Bolivia, were collected in 2010 and 2011 from one site in Monte Negro, which is 150 miles South/Southeast of Porto Velho, the capital of Rondonia. Lastly, sample collection in the state of Para State, located in the eastern end of the Brazilian Amazon basin in northern Brazil, occurred in 2011 and 2012 at two sites. These sites were Goianesia do Para, which is in the eastern portion of Para, and Itaituba, which is on the western side of the state. The two collection sites in Bolivia were at clinics in Riberalta and Guajara-Mirim, which borders Rondonia, Brazil in 2010. Collection sites are shown on Fig 1.

### Sample collection

We included febrile patients 5 years or older (excluding pregnant women) with laboratory-confirmed *P*. *falciparum* mono-infection. In Brazil, *P*. *falciparum* infection was confirmed by either microscopy (Para and Acre states) or by a non–PfHRP2-based RDT (CareStart Malaria, ACCESSBIO, New Jersey, USA) that detected *P*. *falciparum*-specific pLDH as well as pan species pLDH (Rondonia state). All patients with *P*. *falciparum* infection presenting during the collection period were interviewed, to collect demographic information and travel history, and had 3 mL of blood collected in an EDTA tube by venipuncture after written informed consent was obtained. Slides for a confirmatory Giemsa-stained thick and thin blood smears was prepared in accordance with recommendations from the Brazilian Ministry of Health. Asexual parasites were counted in 100 microscopic fields at 100x objective. Final asexual parasitemia was calculated with the assumption that 100 microscopic fields are equivalent to 0.2 μL of blood [24]. The sexual forms (gametocytes) were also counted and their number was estimated per μL of blood. The thin smear was used to confirm species identification. In Rondonia, Brazil, we collected the results of malaria RDT performed at health posts. We also collected information on microscopy results done by routine services for consideration in those cases the expert microscopy could not be obtained at the Instituto Evandro Chagas (IEC) in Belem, Brazil due to lost or damaged slides. Information on patients and laboratory results in Bolivia were more limited. For the sites in Bolivia, parasitemia was not available but all patients were determined to have *P*. *falciparum* monoinfection according to Bolivian Ministry of Health procedures using routine microscopy.

The 3mL venous blood collected in EDTA tubes from each patient was split into three aliquots. Aliquots were centrifuged to separate blood cells (pellets) from clear plasma. Pellets and plasma were separated and transferred to polypropylene tubes for storage at -20°C until laboratory analysis could be performed. All specimens collected in Brazil were sent to the IEC in Para State for sample preparation and analysis. The DNA prepared from the blood pellet of one aliquot from each specimen, as well as aliquots of the corresponding plasma samples, were sent to the Malaria Laboratory Research and Development Unit at the US Centers for Disease Control and Prevention (CDC) in Atlanta for *pfhrp2* and *pfhrp3* molecular genetic studies and PfHRP2 detection, respectively, while another DNA aliquot was also processed at the IEC in Para State for the molecular genetic studies. We rectified any discrepancies in the results obtained between the IEC and CDC laboratories by further independent analysis conducted at the CDC laboratories. Specimens collected in Bolivia (whole blood and blood spots on Whatman 903 filter paper) were shipped to CDC in Atlanta for *pfhrp2* and *pfhrp3* molecular genetic analysis only; no antigen detection was conducted in these samples.

### DNA extraction and PCR

The genomic DNA used for molecular genotyping was extracted from blood pellets and filter paper using Qiagen DNA extraction kits (QIAGEN USA, Valencia, CA) at IEC. We extracted DNA from 200 μL of whole blood, which was eluted into 200 μL of sterile water. Two microliters of DNA from this stock was used in our PCR experiments. DNA quality was assessed by amplifying the merozoite surface protein 2 (*msp2*) using the nested primers described by Akinyi et al. (2013) for 45 cycles and *18S rRNA* [22] genes by nested PCR as previously described [16]. The nested PCR method was found to detect 1–2 parasites/μl of blood as evaluated in our laboratory. *Pfhrp2* and *pfhrp3* genes, along with their respective flanking genes were then amplified in samples in which both *18S rRNA* and *msp2* had been successfully amplified. The primers and conditions for *pfhrp2* and *pfhrp3* gene amplification have been reported previously [16, 17].

For all PCR experiments, when successful amplification of a target gene was accomplished, that result was treated as evidence for the presence of the target gene and the findings were scored as the final test result. When a gene failed to be amplified, we repeated PCR for confirmation. If the result from the second amplification was concordant with the first result indicating no gene product amplification, the result was scored as negative. However, if the second result was discordant with the first, the PCR was performed a third time and the two matching results out of three were scored as the final result. We included three *P*. *falciparum* laboratory strains as controls for genotyping analysis: 3D7, which is positive for *pfhrp2* and *pfhrp3* gene; Dd2, which is negative for *pfhrp2* gene and positive for *pfhrp3;* and HB3, which is positive for *pfhrp2* and negative for *pfhrp3*.

### PfHRP2 antigen

The PfHRP2 antigen concentration in plasma was measured using the SD Malaria Antigen ELISA kit (Standard Diagnostics, Inc., Kyonggi-do, Korea) according to the manufacturer’s instructions. We used plasma samples for the detection of PfHRP2 by ELISA. Briefly, we mixed 150 μL of PBS solution containing mouse anti-PfHRP2 antibody conjugated to horseradish peroxidase (HRPO) with 100 μL of diluted plasma samples to be tested, including standards and negative controls. Following a 30-minute incubation, 100 μL of this mix was transferred to microtiter plates coated with mouse anti-PfHRP2 specific antibodies and incubated for 90 min at room temperature. The plates were washed six times with buffer supplied in the ELISA kit. Finally, 100 μL of hydrogen peroxidase tetramethyl benzidine was then added, followed by incubation at room temperature for 10 minutes in the dark. The reaction was stopped by adding 100 μL of hydrochloric acid and read spectrometrically at 450 nm using the SoftMax Pro 4.8 microplate software and reader (Molecular Devices, Sunnyvale, CA).

In all plates, eight different serial dilutions ranging from 0.39 ng/mL to 50 ng/mL of purified recombinant PfHRP2 were run in duplicate to construct a standard curve. This reference standard curve was used for determining the concentration of PfHRP2 in test samples. The negative controls included plasma from five malaria-uninfected USA donors. Cut-off values for each run were calculated as the mean absorbance of the negative control samples plus 0.100. The OD range in negative controls varied from 0.107 to 0.165. Each plasma sample was diluted 1:5 to 1:20, when necessary, in PBS-Tween and tested in duplicate. We estimated concentrations of PfHRP2 by plotting OD values in a plasma standard curve graph obtained using the recombinant protein standard curve.

### Statistical analysis

Data for statistical analysis were entered into Epi Info software (version 3.5.1, Centers for Disease Control and Prevention, Atlanta, USA). Ninety-five percent confidence intervals were calculated for the proportion of gene deletions for each site of collection using the SAS program (version 9.3, SAS Institute, Cary, NC). It is important to consider that these are not to be considered population-based estimates due to limitations in the sample collection and evaluation design. We used chi square and Spearman’s test to evaluate relationship among variables as appropriate. Since 95% confidence intervals for proportions were calculated for each geographical area only, no clustering effect was accounted for.

### Ethics statement

This molecular surveillance study to determine the occurrence of *P*. *falciparum* parasite isolates with deletions of *pfhrp2* and *pfhrp3* genes, along with their respective flanking genes was approved by both local state (CEP/IEC/SVS/MS #033/2011) and national ethical committees in Brazil (CONEP # 434/2011). This protocol was also approved by the Bolivian National Ethical Committee. The study protocol was also evaluated by CDC’s Human Subjects’ Research office and approved as a non-research activity for surveillance purposes (#990192). We obtained written informed consent from all adult participants and from the parents/ guardians of children.

## Results

A total of 212 DNA and plasma specimens were collected from *P*. *falciparum*-infected patients in Brazil between 2010 and 2012 from Acre, Para, and Rondonia states. These were sent to the IEC and CDC malaria laboratories for genetic analyses, while quantitative ELISA of plasma for PfHRP2 concentrations was conducted only at CDC. A total of 198 specimens met the inclusion criteria for this study by yielding positive results for the amplification of both *18S rRNA* and *msp2* genes and were, therefore, considered valid for further molecular analysis. Seventy-nine (39.9%) *P*. *falciparum* samples were collected in Acre, 59 (29.8%) in Para and 60 (30.3%) in Rondonia.

Among the 198 Brazilian participants whose samples met the inclusion criteria, the median age was 30 years (range: 6–77). Among participants for whom information on sex was available, the majority were male (88 patients, 64.7%). Thirty-three (17.4%) participants reported travel to a city other than that of the collection site in the four weeks prior to sample collection. The geometric mean density of asexual parasitemia among Brazilian patients included in our study was 301.1 parasites/uL (range: 0–62,500), while the arithmetic mean of gametocytemia was 68.5 parasites/uL (range: 0–500).

In total, we found 27 samples to be *pfhrp2* negative with a parasitemia ranging from 10–8,000 parasites/μl; 25 of them from Acre Stateand 2 from Rondonia. Similarly, we found 71 samples to be *pfhrp3* negative with parasitemia ranging from 10–10,000 parasites/μl; 30 negative samples in Acre and Para states each and 11 in Rondonia (S1 Table). These results are summarized in Table 1. A total of 23 double negative for *pfhrp2* and *pfhrp3* were detected in Acre (21 samples) and Rondonia (2 samples). We determined that among the genes flanking *pfhrp2*, PF3D7_0831900, which is located on the 5’ side of *pfhrp2*, had the highest deletion frequency, 42 occurrences, while only one sample (in Rondonia) had deleted the gene located on the 3’ end of *pfhrp2*, PF3D7_0831700 when all three sites in Brazil are considered (Table 2). Meanwhile, 35 isolates had deleted PF3D7_1372400, which is the gene located on the 5’ side of *pfhrp3*, while 41 samples were lacking the gene flanking *pfhrp3* on the 3’ end, PF3D7_1372100 (Table 2). Table 3 shows the number of isolates collected in Brazil with different combinations of *pfhrp2* and *pfhrp3* genes, along with their respective flanking genes.

**Table 1:** *Pfhrp2* and *pfhrp3* gene deletions among samples collected in Brazil (198 samples) and Bolivia (25 samples).

Site	*Pfhrp2* deletion		*Pfhrp3* deletion		Total
	N (%)	95% confidence interval	N (%)	95% confidence interval	
Acre, Brazil	25/79 (31.6%)	21.6–43.1	30/79 (38.0%)	27.3–49.6	79
Para, Brazil	0/59 (0%)	NA	30/59 (50.9%)	37.5–64.1	59
Rondonia, Brazil	2/60 (3.3%)	0.4–11.5	11/60 (18.3%)	9.5–30.4	60
Bolivia	1/25 (4.0%)	0.1–20.4	17/25 (68.0%)	46.5–85.1	25

**Table 2:** Deletions of *pfhrp2* and *pfhrp3* flanking genes in samples from Brazil (198 samples) and Bolivia (25 samples).

Site	*Pfhrp2* gene		*Pfhrp3* gene		Total
	Pf3D7_0831700 deletion, n (%) 95% CI	Pf3D7_0831900 deletion, n (%) 95% CI	Pf3D7_1372100 deletion, n (%) 95% CI	Pf3D7_1372400 deletion, n (%) 95% CI	
Acre, Brazil	0/79 (0%) (NA)	35/79 (44.3%) (33.1–55.9%)	21/79 (28.8%) (18.8–40.6%)*	15/79 (19.5%) (10.6–28.3%)**	79
Para, Brazil	0/59 (0%) (NA)	1/59 (1.7%) (0–9.1%)	12/59 (25.0%) (12.8–37.3%)^$^	12/59 (20.7%) (11.2–33.4%)^$ $^	59
Rondonia, Brazil	1/60 (1.7%) (0–8.9%)	6/60 (10.0%) (3.8–20.5%)	8/60 (13.3%) (4.7–21.9%)	8/60 (13.3%) (5.9–24.6%)	60
Bolivia	9/25 (36.0%) (18.0–57.5%)	0/25 (0%) NA	12/25 (48.0%) (27.8–68.7%)	14/25 (56.0%) (34.9–75.6%)	25

Note:

* 6 missing;

** 2 missing;

^$^ 11 missing;

^$ $^ 1 missing;

NA = Not available.

**Table 3:** Combination of *Pfhrp2* and *Pfhrp3* genes and respective flanking genes in *P*. *falciparum* samples collected in Brazil (198 samples).

Gene	*Pfhrp2* gene				*Pfhrp3* gene			
	Pf3D7_0831900	*Pfhrp2*	Pf3D7_0831700	N	Pf3D7_1372400	*Pfhrp3*	Pf3D7_1372100	N
	Present	Present	Present	154	Present	Present	Present	115
	Deleted	Present	Present	17	Deleted	Present	Present	7
	Present	Deleted	Present	2	Present	Deleted	Present	7
	Deleted	Deleted	Present	24	Deleted	Deleted	Present	9
	Deleted	Deleted	Deleted	1	Deleted	Deleted	Deleted	16
					Present	Deleted	Deleted	25
					Insufficient data			19
Total				198				198

A total of 27 specimens were collected in Bolivia and 25 of these met the inclusion criteria when analyzed at CDC. Of these 25 valid Bolivia samples, only one (4%) was found to have deleted the *pfhrp2* gene, while 17 (68%) isolates had deleted *pfhrp3* (Table 1). Although only 1 specimen was found to have deleted *pfhrp2*, 9 (36%) of the isolates had deleted the flanking PHIST family gene PF3D7_0831900 (Table 2). PF3D7_1372100, which flanks *pfhrp3*, was absent in 12 isolates (48%), while the flanking gene, PF3D7_1372400, which flanks 3’ of *pfhrp3*, was deleted in 14 (56%) isolates (Table 2).

### PfHRP2 antigen detection

Of the 198 Brazilian plasma specimens, 102 had detectable PfHRP2 levels in plasma with only two of those samples being negative for *pfhrp2* gene (both from Acre), while among the 95 samples with no detectable levels of PfHRP2, 25 were negative for *pfhrp2*. One sample did not have valid results for HRP2 protein. There was an association between *pfhrp2* gene and PfHRP2 in these patients (chi square = 24.7, p <0.0001). However, among 170 samples positive for the *pfhrp2* gene, 70 (41.2%) had undetectable levels of PfHRP2 in the plasma. Of the 170 *pfhrp2* Brazilian samples positive for the *pfhrp2* gene, 100 of the corresponding plasma specimens were also positive for PFHRP2, with concentrations varying from 0.4 to 674.1 ng/ml. These results stratified by collection site in Brazil are presented in Table 4.

**Table 4:** Number of samples positive for *P*. *falciparum* histidine-rich protein 2 (PfHRP2) protein and protein level among for *pfhrp2* gene positive specimens, Brazil (170 samples).

Site	PfHRP2, n (%) (95% CI)	Plasma PfHRP-2, median (ng/mL) (range)	*Pfhrp2* gene–positive samples, n
Acre, Brazil	31 (57.4%) (44.2–70.6%)	37.1 (2.4–395.7)	54
Para, Brazil	27 (46.6%) (33.7–59.4%)	24.9 (0.7–343.9)	58*
Rondonia, Brazil	42 (72.4%) (60.9–83.9%)	63.2 (0.4–674.1)	58
Total	100**	35.6 (0.4–674.1)	170*

Note:

* 1 missing;

** percentage not calculated due to design constraint

Considering all samples, a correlation was found between PfHRP2 levels and parasitemia when asexual parasitemia and gametocytemia were considered together (Spearman’s correlation coefficient = 0.3392, p< 0.0001). The percentage of the *pfhrp2-*positive samples that were also positive for PfHRP2 was highest in Rondonia (72.4%), followed by the states of Acre (61.6%) and Para (45.8%). The difference in the proportion of specimens positive for *pfhrp2* gene and PfHRP2 was statistically significant across the collection sites (*p* = 0.003).

## Discussion

The findings from this study confirm that *P*. *falciparum* parasite populations with deletions of the *pfhrp2* and *pfhrp3* genes are present in Brazil and Bolivia. The highest proportion of *pfhrp2*-negative isolates (31.2%) was found in Cruzeiro do Sul municipality in Acre state, Brazil. This relatively high percentage of isolates with *pfhrp2*-deleted parasites is comparable to what has been observed previously in the neighboring Peruvian Amazon River basin, where the prevalence of *pfhrp2*-negative parasites has been reported to be between 30 and 40% [16, 17]. The specimens collected in Monte Negro, Rondonia State, had a much lower percentage of isolates with deletion of the *pfhrp2* gene (3.3%). The proportion of *P*. *falciparum* isolates with deletion of *pfhrp2* gene was also low (4%) in the Department of Beni in Bolivia, which borders Rondonia. In Para state, Brazil, which is situated at the eastern end of the Amazon River basin, no parasites (out of 59 samples) were detected with *pfhrp2* deletions. Although these data may seem to suggest that *pfhrp2*-negative parasites are confined to the western border regions of Brazil, our study was not designed to provide representative information about the distribution or prevalence of *pfhrp2* and *pfhrp3* genetic variants. Given that the three states we surveyed accounted for approximately 56% of the reported cases of *P*. *falciparum* in the Brazilian Amazon Basin in 2012 [Brazilian National Reportable Disease Information System for Malaria (SIVEP)], a more comprehensive and representative survey should be conducted to evaluate *pfhrp2* gene deletion in these 3 states as well as Amazonas, Roraima, and Amapa.

Thus far, the proportion of *pfhrp2*-negative parasite isolates in this region of Acre is comparable to findings from other studies in South America. In Peru (Loreto Department) and Colombia (Amazonas Department), 41% and 67% of *P*. *falciparum* parasite isolates were found to be *pfhrp2* negative, respectively [17]. Reports from other countries and sites indicate that isolates with deletions in *pfhrp2* and *pfhrp3* genes have not been detected or were relatively rare [20–22]. Taken together, these reports suggest *pfhrp2*-negative parasites may be concentrated on the contiguous Amazon River Basin areas of Loreto Department, Acre State, and the Amazonas Department in Peru, Brazil and Colombia, respectively. In view of this, it would not be unreasonable to suspect that the western portion of the state of Amazonas in Brazil will also have a substantial proportion of *pfhrp2-*negative parasites. Outside of this core region, it appears that the vast majority of *P*. *falciparum* parasites have an intact *pfhrp2* gene and are, therefore, likely to be detected by PfHRP2-based RDTs. These findings and considerations underscore the need to implement board, appropriate, and representative surveillance systems to monitor *pfhrp2* gene deletions in Amazon region.

Considering that *pfhrp2* and *pfhrp3* genes are located on different chromosomes (8 and 13, respectively), the deletions of these two paralogous genes are likely to be independent events. At this time we do not have a clear understanding of why segments of the genome where *pfhrp2* and *pfhrp3* are located are subject to deletion. Understanding this biological process will be essential to determining if there is any link between the deletions of these two genes that are located on different chromosomes. However, this assumption of independence may not be a completely solid indication that the deletion of one gene does not influence the eventual deletion of the other. There was a higher proportion of *pfhrp3* deletions compared to *pfhrp2* deletions among parasite isolates collected in all three Brazilian States. The proportion of *pfhrp3*-negative isolates was similarly much higher in Bolivia (68%) compared to that of *pfhrp2*-negative parasites. This trend has also been observed in other studies conducted in Peru, Colombia, and Honduras [16, 17, 19, 25]. The factors that drive both the gene deletions and the expansion of *pfhrp2-* or *pfhrp3-*negative parasites in *P*. *falciparum* populations in these areas are not known [16, 17, 26]. For this reason, statistically sound and representative surveillance or periodic surveys of parasite populations in Brazil and other areas of South and Central America for expression of *pfhrp2* and *pfhrp3* gene is strongly recommended.

In this study, PfHRP2 antigen concentration was measured in plasma samples from *P*. *falciparum-*infected patients who had different combinations of *pfhrp2* and *pfhrp3* gene deletions, primarily as an ancillary method to confirm *pfhrp2* gene expression. We detected PfHRP2 in the plasma in most, but not all, samples that were shown to have an intact *pfhrp2* gene by molecular methods. We showed that PfHRP2 concentration levels in the plasma has a correlation, albeit weak, with parasite levels obtained by microscopy including both sexual and asexual parasites. Likewise, studies carried out in Tanzania and Papua New Guinea, areas of high malaria transmission, have shown that PfHRP2 plasma levels correlated with log *P*. *falciparum* parasitemia density in children [27, 28]. This supports the value of RDTs, which have detection limit of 200 parasites/μL, to detect active clinical malaria infections, since patients with higher parasitemia will have greater chances of being detected as positives.

As for the plasma samples negative for PfHRP2 antigen among the *pfhrp2* positive specimens, there are various potential biological and technical explanations for the inability to detect PfHRP2 in the plasma of these specimens. First, PfHRP2 varies in expression during blood stage development; the most significant increase in PfHRP2 levels occurs in the late ring and trophozoite stages and accumulates variably in the plasma during the course of infection [10, 29, 30]. Second, high antibody titers may be produced by the host, and immune complexes with anti-PfHRP2 antibodies can either accelerate PfHRP2 clearance or impair the ability to detect PfHRP2 antigens in ELISA or RDTs by blocking epitopes recognized by the capture or detection antibodies in these assays [31]. Thus, only attempting to detect PfHRP2in plasma can be variable as the most significant reservoir of PfHRP2 antigen is in the infected erythrocyte. Two *pfhrp2* negative isolates collected in Acre apparently had detectable levels of PfHRP2 antigen in the corresponding plasma specimens. However, those two blood samples also were *pfhrp3*-positive and, therefore, were able to produce PfHRP3 protein. PfHRP3 can share similar repeat epitopes with PfHRP2, that can cross-react with some monoclonal antibodies and give positive reactions in PfHRP2 ELISA capture assays [14, 15] and with some RDTs. For this reason, the PfHRP2 ELISA or other serological capture assays can show positive results in *pfhrp2*-negative/ *pfhrp3*-positive samples when sufficient quantities of PfHRP3 antigen are present.

This study has several important limitations. Firstly, our study findings are limited to the samples we collected in a limited number of sites at relatively short (and different) times over the last 5 years. Our findings should not be generalized to Acre, Rondonia, or Para States, Beni Department, or other areas of the Brazilian or Bolivian Amazon. Therefore, systematic sample collection in these and other sites of the Amazon region is necessary to evaluate the population prevalence of *pfhrp2* and *pfhrp3* gene deletions. In addition, our sample collection expanded over a few years, from 2010 through 2012. So, our findings were subject to variations along time for these regions. Finally, we were not able to collect complete epidemiological information in Bolivia to allow for inferences about place of residence and travel history for this group of patients. It is possible that the single Bolivian isolate that demonstrated *pfhrp2* deletion was acquired elsewhere.

In summary, this study confirms the presence of *pfhrp2-* and *pfhrp3-*deleted *P*. *falciparum* populations in parts of the Brazilian and Bolivia Amazon regions, similar to what has been observed previously in studies in the Peruvian and Colombian Amazon River basin. Although our study was not designed to evaluate differences between sites nor provide population-based estimates, the proportion of isolates with *pfhrp2* deletions varied across the three different states we surveyed with the highest percentage found in isolates from Acre State. Given our findings, it is critical to reconsider the use of malaria RDTs based exclusively on detection of PfHRP2 in Acre State, where the proportion of *pfhrp2*-negative parasites likely exceeds 30%, and determine more appropriate alternative malaria RDTs or diagnostic assays to employ. It is important to continue monitoring the proportion of *pfhrp2-*deleted *P*. *falciparum* parasites in different geographical locations within the Amazon Basin because, although most parasites east of Acre were *pfhrp2* gene positive, large portions of the parasite populations in those states had deleted the *pfhrp3* gene and the *pfhrp2* positive population structure may not be stable.

## Supporting information

S1 Table Meta data for all samples analyzed in this study. (PDF)
