# Supplementary material for: Histidine-rich protein 2 (pfhrp2) and pfhrp3 gene deletions in Plasmodium falciparum isolates from select sites in Brazil and Bolivia
Source: PLoS One. 2017 Mar 16;12(3):e0171150. doi: 10.1371/journal.pone.0171150 (PMC5354239; doi:10.1371/journal.pone.0171150)
Supplement: S1 Table — (PDF) [file pone.0171150.s001.pdf]

	A	B	C	D	E	F	G	H	I	J	K	L
1	**S1 Table. Meta data for all samples analysed in this study**											
2												
3	Study site/State	Sample ID	Parasitemia/ microliter (Asexual stage)	Parasitemia per microliter (Sexual stage)	*18S rRNA*	*msp2*	PF3D7_0831900	*pfhrp2* (Exon 1-2)	PF3D7_0831700	PF3D7_1372400	*pfhrp3* (Exon 1-2)	PF3D7_1372100
4	Para	HRP2 15/11	100	0	Pos	Pos	Pos	Pos	Pos	Pos	Neg	Neg
5	Para	HRP2 16/11	0	150	Pos	Pos	Pos	Pos	Pos	Neg	Neg	Neg
6	Para	HRP2 17/11	0	5	Pos	Pos	Neg	Pos	Pos	Neg	Neg	Neg
7	Para	HRP2 18/11	6000	7500	Pos	Pos	Pos	Pos	Pos	Pos	Neg	no sample for testing
8	Para	HRP2 19/11	215	0	Pos	Pos	Pos	Pos	Pos	Pos	Neg	Neg
9	Para	HRP2 20/11	25	0	Pos	Pos	Pos	Pos	Pos	Neg	Neg	Neg
10	Para	HRP2 21/11	6000	0	Pos	Pos	Pos	Pos	Pos	Pos	Neg	Neg
11	Para	HRP2 22/11	2000	0	Pos	Pos	Pos	Pos	Pos	Pos	Neg	no sample for testing
12	Para	HRP2 23/11	230	0	Pos	Pos	Pos	Pos	Pos	Pos	Neg	Neg
13	Para	HRP2 24/11	1500	0	Pos	Pos	Pos	Pos	Pos	Pos	Neg	Neg
14	Para	HRP2 25/11	500	0	Pos	Pos	Pos	Pos	Pos	Pos	Neg	Neg
15	Para	HRP2 26/11	200	100	Pos	Pos	Pos	Pos	Pos	Pos	Neg	no sample for testing
16	Para	HRP2 27/11	1500	0	Pos	Pos	Pos	Pos	Pos	Pos	Neg	no sample for testing
17	Para	HRP2 28/11	100	100	Pos	Pos	Pos	Pos	Pos		Neg	no sample for testing
18	Para	HRP2 29/11	3500	0	Pos	Pos	Pos	Pos	Pos	Pos	Neg	no sample for testing
19	Para	HRP2 30/11	6000	100	Pos	Pos	Pos	Pos	Pos	Pos	Neg	Neg
20	Para	HRP2 31/11	500	0	Pos	Pos	Pos	Pos	Pos	Pos	Neg	no sample for testing
21	Para	HRP2 32/11	10	0	Pos	Pos	Pos	Pos	Pos	Pos	Neg	no sample for testing
22	Para	HRP2 33/11	500	0	Pos	Pos	Pos	Pos	Pos	Pos	Neg	no sample for testing
23	Para	HRP2 34/11	50	0	Pos	Pos	Pos	Pos	Pos	Pos	Pos	Pos
24	Para	HRP2 35/11	300	0	Pos	Pos	Pos	Pos	Pos	Pos	Pos	Pos
25	Para	HRP2 36/11	300	0	Pos	Pos	Pos	Pos	Pos	Neg	Neg	Pos
26	Para	HRP2 37/11	3500	0	Pos	Pos	Pos	Pos	Pos	Pos	Pos	Pos
27	Para	HRP2 38/11	8000	0	Pos	Pos	Pos	Pos	Pos	Pos	Pos	Pos
28	Para	HRP2 39/11	26000	0	Pos	Pos	Pos	Pos	Pos	Pos	Pos	Pos
29	Para	HRP2 40/11	200	0	Pos	Pos	Pos	Pos	Pos	Pos	Pos	Pos
30	Para	HRP2 41/11	62500	0	Pos	Pos	Pos	Pos	Pos	Pos	Pos	Pos
31	Para	HRP2 42/11	150	0	Pos	Pos	Pos	Pos	Pos	Pos	Pos	Pos
32	Para	HRP2 43/11	50	0	Pos	Pos	Pos	Pos	Pos	Neg	Neg	Pos
33	Para	HRP2 44/11	10000	0	Pos	Pos	Pos	Pos	Pos	Neg	Pos	Pos
34	Para	HRP2 45/11	10000	0	Pos	Pos	Pos	Pos	Pos	Pos	Pos	Pos
35	Para	HRP2 46/11	200	0	Pos	Pos	Pos	Pos	Pos	Pos	Pos	no sample for testing
36	Para	HRP2 47/11	200	0	Pos	Pos	Pos	Pos	Pos	Pos	Pos	Pos
37	Para	HRP2 48/11	2000	0	Pos	Pos	Pos	Pos	Pos	Pos	Pos	Pos
38	Para	HRP2 49/12	5000	0	Pos	Pos	Pos	Pos	Pos	Pos	Pos	Pos
39	Para	HRP2 50/12	20000	0	Pos	Pos	Pos	Pos	Pos	Pos	Pos	Pos
40	Para	HRP2 51/12	60	0	Pos	Pos	Pos	Pos	Pos	Pos	Pos	Pos
41	Para	HRP2 52/12	5000	0	Pos	Pos	Pos	Pos	Pos	Pos	Pos	Pos
42	Para	HRP2 53/12	40	0	Pos	Pos	Pos	Pos	Pos	Neg	Neg	Pos
43	Para	HRP2 54/12	12500	0	Pos	Pos	Pos	Pos	Pos	Pos	Pos	Pos
44	Para	HRP2 55/12	10000	10	Pos	Pos	Pos	Pos	Pos	Pos	Pos	Pos
45	Para	HRP2 56/12	3000	0	Pos	Pos	Pos	Pos	Pos	Pos	Pos	Pos
46	Para	HRP2 57/12	9000	0	Pos	Pos	Pos	Pos	Pos	Pos	Neg	Pos
47	Para	HRP2 58/12	150	0	Pos	Pos	Pos	Pos	Pos	Pos	Neg	Pos
48	Para	HRP2 59/12	0	25	Pos	Pos	Pos	Pos	Pos	Neg	Neg	Pos
49	Para	HRP2 60/12	125	0	Pos	Pos	Pos	Pos	Pos	Neg	Neg	Neg
50	Para	HRP2 61/12	1000	0	Pos	Pos	Pos	Pos	Pos	Neg	Neg	Neg
51	Para	HRP2 62/12	2000	0	Pos	Pos	Pos	Pos	Pos	Pos	Pos	Pos
52	Para	HRP2 63/12	0	20	Pos	Pos	Pos	Pos	Pos	Neg	Neg	no sample for testing
53	Para	HRP2 64/12	0	10	Pos	Pos	Pos	Pos	Pos	Neg	Neg	Pos
54	Para	HRP2 65/12	100	40	Pos	Pos	Pos	Pos	Pos	Pos	Neg	Pos
55	Para	HRP2 66/12	1000	0	Pos	Pos	Pos	Pos	Pos	Pos	Pos	Pos

	A	B	C	D	E	F	G	H	I	J	K	L
56	Para	HRP2 67/12	4000	0	Pos	Pos	Pos	Pos	Pos	Pos	Pos	Pos
57	Para	HRP2 69/12	10000	0	Pos	Pos	Pos	Pos	Pos	Pos	Pos	Pos
58	Para	HRP2 70/12	25	0	Pos	Pos	Pos	Pos	Pos	Pos	Pos	Pos
59	Para	HRP2 71/12	5000	0	Pos	Pos	Pos	Pos	Pos	Pos	Pos	Pos
60	Para	HRP2 72/12	400	0	Pos	Pos	Pos	Pos	Pos	Pos	Pos	Pos
61	Para	HRP2 73/12	600	0	Pos	Pos	Pos	Pos	Pos	Pos	Pos	Pos
62	Para	HRP2 74/12	1500	0	Pos	Pos	Pos	Pos	Pos	Pos	Pos	Pos
63	Acre	BRA-A 001	no data	no data	Pos	Pos	Pos	Pos	Pos	Neg	Pos	Pos
64	Acre	BRA-A 002	no data	no data	Pos	Pos	Pos	Pos	Pos	Pos	Pos	Pos
65	Acre	BRA-A 003	420	0	Pos	Pos	Neg	Neg	Pos	Pos	Neg	Neg
66	Acre	BRA-A 004	0	130	Pos	Pos	Pos	Pos	Pos	Pos	Pos	Pos
67	Acre	BRA-A 005	500	no data	Pos	Pos	Neg	Neg	Pos	Pos	Neg	Neg
68	Acre	BRA-A 007	0	30	Pos	Pos	Neg	Neg	Pos	Pos	Neg	Neg
69	Acre	BRA-A 008	25	no data	Pos	Pos	Pos	Pos	Pos	Pos	Pos	Pos
70	Acre	BRA-A 010	30	no data	Pos	Pos	Pos	Pos	Pos	Pos	Pos	Pos
71	Acre	BRA-A 012	20	0	Pos	Pos	Neg	Neg	Pos	Pos	Neg	Neg
72	Acre	BRA-A 013	5000	0	Pos	Pos	Neg	Neg	Pos	Pos	Neg	Neg
73	Acre	BRA-A 014	50	0	Pos	Pos	Pos	Pos	Pos	Pos	Pos	Pos
74	Acre	BRA-A 015	25	0	Pos	Pos	Neg	Neg	Pos	Neg	Neg	Neg
75	Acre	BRA-A 016	60	0	Pos	Pos	Pos	Pos	Pos	Pos	Pos	Pos
76	Acre	BRA-A 017	500	0	Pos	Pos	Pos	Pos	Pos	Neg	Neg	no sample for testing
77	Acre	BRA-A 019	500	0	Pos	Pos	Pos	Pos	Pos	Pos	Neg	Pos
78	Acre	BRA-A 021	8000	0	Pos	Pos	Neg	Neg	Pos	Pos	Neg	Neg
79	Acre	BRA-A 022	500	400	Pos	Pos	Neg	Neg	Pos	Pos	Neg	Neg
80	Acre	BRA-A 024	80	0	Pos	Pos	Neg	Neg	Pos	Neg	Neg	Neg
81	Acre	BRA-A 026	10	15	Pos	Pos	Neg	Neg	Pos	Neg	Neg	Neg
82	Acre	BRA-B 001	11000	0	Pos	Pos	Pos	Pos	Pos	Pos	Pos	Pos
83	Acre	BRA-B 002	no data	no data	Pos	Pos	Pos	Pos	Pos	Pos	Pos	Pos
84	Acre	BRA-B 003	500	0	Pos	Pos	Pos	Pos	Pos		Pos	Pos
85	Acre	BRA-B 004	10	no data	Pos	Pos	Pos	Pos	Pos		Pos	Pos
86	Acre	BRA-B 005	25	10	Pos	Pos	Pos	Pos	Pos	Pos	Pos	Pos
87	Acre	BRA-B 006	500	10	Pos	Pos	Neg	Pos	Pos	Pos	Pos	Pos
88	Acre	BRA-B 007	45	0	Pos	Pos	Neg	Pos	Pos	Neg	Pos	Pos
89	Acre	BRA-B 009	350	0	Pos	Pos	Neg	Pos	Pos	Pos	Pos	Pos
90	Acre	BRA-B 010	110	0	Pos	Pos	Neg	Pos	Pos	Pos	Pos	Pos
91	Acre	BRA-B 011	4000	0	Pos	Pos	Pos	Pos	Pos	Pos	Pos	Pos
92	Acre	BRA-B 012	1000	0	Pos	Pos	Neg	Neg	Pos	Pos	Neg	Neg
93	Acre	BRA-B 013	300	0	Pos	Pos	Neg	Neg	Pos	Neg	Neg	Neg
94	Acre	BRA-B 014	15	0	Pos	Pos	Neg	Neg	Pos	Pos	Neg	Neg
95	Acre	BRA-B 015	15	0	Pos	Pos	Neg	Pos	Pos	Neg	Neg	Pos
96	Acre	BRA-B 016	60	0	Pos	Pos	Neg	Pos	Pos	Neg	Pos	Pos
97	Acre	BRA-B 017	200	0	Pos	Pos	Neg	Neg	Pos	Pos	Neg	no sample for testing
98	Acre	BRA-B 018	1500	0	Pos	Pos	Pos	Pos	Pos	Pos	Pos	Pos
99	Acre	BRA-C 001	60	0	Pos	Pos	Neg	Neg	Pos	Pos	Neg	Neg
100	Acre	BRA-C 002	300	no data	Pos	Pos	Neg	Neg	Pos	Pos	Neg	Neg
101	Acre	BRA-C 003	0	160	Pos	Pos	Pos	Pos	Pos	Pos	Pos	Pos
102	Acre	BRA-C 004	300	no data	Pos	Pos	Pos	Pos	Pos	Pos	Pos	Pos
103	Acre	BRA-C 005	no data	no data	Pos	Pos	Pos	Pos	Pos	Pos	Pos	Pos
104	Acre	BRA-C 006	no data	no data	Pos	Pos	Neg	Pos	Pos	Pos	Pos	Pos
105	Acre	BRA-C 007	0	1000	Pos	Pos	Neg	Pos	Pos	Pos	Pos	Pos
106	Acre	BRA-C 008	no data	no data	Pos	Pos	Pos	Pos	Pos	Pos	Pos	Pos
107	Acre	BRA-C 009	0	500	Pos	Pos	Pos	Pos	Pos	Pos	Pos	Pos
108	Acre	BRA-C 011	500	0	Pos	Pos	Pos	Pos	Pos	Pos	Pos	Pos
109	Acre	BRA-C 012	5500	0	Pos	Pos	Pos	Pos	Pos	Pos	Pos	Pos
110	Acre	BRA-C 013	135	0	Pos	Pos	Neg	Pos	Pos	Pos	Pos	Pos
111	Acre	BRA-C 014	1000	0	Pos	Pos	Neg	Neg	Pos	Pos	Neg	Neg
112	Acre	BRA-C 016	100	0	Pos	Pos	Pos	Pos	Pos	Pos	Pos	Pos

	A	B	C	D	E	F	G	H	I	J	K	L
113	Acre	BRA-C 017	7500	0	Pos	Pos	Pos	Pos	Pos	Pos	Pos	Pos
114	Acre	BRA-C 018	1000	35	Pos	Pos	Pos	Pos	Pos	Pos	Pos	Pos
115	Acre	BRA-C 019	3000	0	Pos	Pos	Pos	Pos	Pos	Pos	Pos	Pos
116	Acre	BRA-C 020	1000	10	Pos	Pos	Pos	Pos	Pos	Pos	Pos	Pos
117	Acre	BRA-C 021	130	0	Pos	Pos	Pos	Neg	Pos	Pos	Neg	no sample for testing
118	Acre	BRA-C 022	65	0	Pos	Pos	Pos	Pos	Pos	Pos	Pos	Pos
119	Acre	BRA-C 023	150	0	Pos	Pos	Pos	Pos	Pos	Pos	Pos	Pos
120	Acre	BRA-C 025	60	0	Pos	Pos	Neg	Pos	Pos	Pos	Neg	Neg
121	Acre	BRA-C 026	120	0	Pos	Pos	Pos	Pos	Pos	Pos	Pos	Pos
122	Acre	BRA-C 027	1000	0	Pos	Pos	Pos	Pos	Pos	Pos	Pos	Pos
123	Acre	BRA-C 028	150	160	Pos	Pos	Pos	Pos	Pos	Neg	Neg	Pos
124	Acre	BRA-C 029	450	0	Pos	Pos	Pos	Pos	Pos	Pos	Pos	no sample for testing
125	Acre	BRA-C 030	3000	0	Pos	Pos	Pos	Pos	Pos	Neg	Neg	Pos
126	Acre	BRA-C 031	60	50	Pos	Pos	Neg	Neg	Pos	Pos	Neg	Neg
127	Acre	BRA-C 032	100	25	Pos	Pos	Pos	Neg	Pos	Neg	Pos	no sample for testing
128	Acre	BRA-C 033	50	0	Pos	Pos	Pos	Pos	Pos	Pos	Pos	Pos
129	Acre	BRA-C 034	3000	0	Pos	Pos	Neg	Neg	Pos	Pos	Pos	Pos
130	Acre	BRA-C 035	500	0	Pos	Pos	Neg	Pos	Pos	Pos	Neg	Pos
131	Acre	BRA-C 036	8000	0	Pos	Pos	Pos	Pos	Pos	Pos	Neg	no sample for testing
132	Acre	BRA-C 037	2000	0	Pos	Pos	Pos	Pos	Pos	Neg	Neg	Neg
133	Acre	BRA-C 038	1500	0	Pos	Pos	Neg	Neg	Pos	Pos	Neg	Neg
134	Acre	BRA-C 039	2500	1500	Pos	Pos	Neg	Pos	Pos	Pos	Pos	Pos
135	Acre	BRA-C 040	2000	0	Pos	Pos	Pos	Pos	Pos	Pos	Pos	Pos
136	Acre	BRA-C 041	1000	0	Pos	Pos	Neg	Neg	Pos	Neg	Neg	Neg
137	Acre	BRA-C 042	100	0	Pos	Pos	Pos	Pos	Pos	Pos	Pos	Pos
138	Acre	BRA-C 043	150	0	Pos	Pos	Pos	Pos	Pos	Neg	Pos	Pos
139	Acre	BRA-C 044	1500	0	Pos	Pos	Pos	Pos	Pos	Pos	Pos	Pos
140	Acre	BRA-C 045	1000	0	Pos	Pos	Neg	Neg	Pos	Pos	Pos	Pos
141	Acre	BRA-C 046	75	0	Pos	Pos	Neg	Neg	Pos	Pos	Pos	Pos
142	Rondonia	BRP 01	900	0	Pos	Pos	Pos	Pos	Pos	Pos	Pos	Pos
143	Rondonia	BRP 02	90	0	Pos	Pos	Pos	Pos	Pos	Pos	Pos	Pos
144	Rondonia	BRP 03	no data	no data	Pos	Pos	Pos	Pos	Pos	Pos	Pos	Pos
145	Rondonia	BRP 04	300	0	Pos	Pos	Pos	Pos	Pos	Pos	Pos	Pos
146	Rondonia	BRP 05	1500	0	Pos	Pos	Pos	Pos	Pos	Pos	Pos	Pos
147	Rondonia	BRP 06	300	0	Pos	Pos	Pos	Pos	Pos	Pos	Pos	Pos
148	Rondonia	BRP 07	300	0	Pos	Pos	Pos	Pos	Pos	Pos	Pos	Pos
149	Rondonia	BRP 08	no data	no data	Pos	Pos	Pos	Pos	Pos	Pos	Pos	Pos
150	Rondonia	BRP 09	no data	no data	Pos	Pos	Pos	Pos	Pos	Pos	Pos	Pos
151	Rondonia	BRP 10	60	0	Pos	Pos	Pos	Pos	Pos	Pos	Pos	Pos
152	Rondonia	BRP 11	90	0	Pos	Pos	Pos	Pos	Pos	Pos	Pos	Pos
153	Rondonia	BRP 12	300	0	Pos	Pos	Pos	Pos	Pos	Pos	Pos	Pos
154	Rondonia	BRP 13	300	0	Pos	Pos	Pos	Pos	Pos	Pos	Pos	Pos
155	Rondonia	BRP 14	90	0	Pos	Pos	Pos	Pos	Pos	Pos	Pos	Pos
156	Rondonia	BRP 15	300	0	Pos	Pos	Pos	Pos	Pos	Pos	Pos	Pos
157	Rondonia	BRP 16	no data	no data	Pos	Pos	Pos	Pos	Pos	Pos	Pos	Pos
158	Rondonia	BRP 17	no data	no data	Pos	Pos	Pos	Pos	Pos	Pos	Pos	Pos
159	Rondonia	BRP 18	150	0	Pos	Pos	Pos	Pos	Pos	Pos	Pos	Pos
160	Rondonia	BRP 19	300	0	Pos	Pos	Pos	Pos	Pos	Pos	Pos	Pos
161	Rondonia	BRP 20	150	0	Pos	Pos	Pos	Pos	Pos	Pos	Pos	Pos
162	Rondonia	BRP 21	150	0	Pos	Pos	Pos	Pos	Pos	Pos	Pos	Pos
163	Rondonia	BRP 22	1200	0	Pos	Pos	Pos	Pos	Pos	Pos	Pos	Pos
164	Rondonia	BRP 23	150	0	Pos	Pos	Neg	Pos	Pos	Pos	Pos	Pos
165	Rondonia	BRP 24	3000	0	Pos	Pos	Pos	Pos	Pos	Pos	Pos	Pos
166	Rondonia	BRP 25	150	0	Pos	Pos	Pos	Pos	Pos	Pos	Pos	Pos
167	Rondonia	BRP 26	150	0	Pos	Pos	Pos	Pos	Pos	Pos	Pos	Pos
168	Rondonia	BRP 27	150	0	Pos	Pos	Pos	Pos	Pos	Pos	Pos	Pos
169	Rondonia	BRP 29	600	0	Pos	Pos	Pos	Pos	Pos	Pos	Pos	Pos

	A	B	C	D	E	F	G	H	I	J	K	L
170	Rondonia	BRP 30	750	0	Pos	Pos	Pos	Pos	Pos	Pos	Pos	Pos
171	Rondonia	BRP 31	30	0	Pos	Pos	Neg	Neg	Neg	Neg	Neg	Neg
172	Rondonia	BRP 33	300	0	Pos	Pos	Neg	Pos	Pos	Neg	Pos	Pos
173	Rondonia	BRP 34	no data	no data	Pos	Pos	Pos	Pos	Pos	Neg	Pos	Pos
174	Rondonia	BRP 35	150	0	Pos	Pos	Pos	Pos	Pos	Pos	Pos	Pos
175	Rondonia	BRP 36	150	0	Pos	Pos	Pos	Pos	Pos	Pos	Pos	Pos
176	Rondonia	BRP 37	60	0	Pos	Pos	Pos	Pos	Pos	Pos	Pos	Pos
177	Rondonia	BRP 38	1200	0	Pos	Pos	Pos	Pos	Pos	Pos	Pos	Pos
178	Rondonia	BRP 39	300	0	Pos	Pos	Pos	Pos	Pos	Pos	Pos	Pos
179	Rondonia	BRP 40	no data	no data	Pos	Pos	Pos	Pos	Pos	Pos	Neg	Neg
180	Rondonia	BRP 41	180	0	Pos	Pos	Pos	Pos	Pos	Pos	Neg	Neg
181	Rondonia	BRP 42	90	0	Pos	Pos	Pos	Pos	Pos	Neg	Neg	Neg
182	Rondonia	BRP 43	1200	0	Pos	Pos	Pos	Pos	Pos	Pos	Pos	Pos
183	Rondonia	BRP 44	no data	no data	Pos	Pos	Neg	Neg	Pos	Pos	Neg	Neg
184	Rondonia	BRP 45	no data	no data	Pos	Pos	Neg	Pos	Pos	Neg	Neg	Neg
185	Rondonia	BRP 46	1200	0	Pos	Pos	Pos	Pos	Pos	Neg	Neg	Neg
186	Rondonia	BRP 47	1200	0	Pos	Pos	Pos	Pos	Pos	Neg	Neg	Neg
187	Rondonia	BRP 48	3000	0	Pos	Pos	Pos	Pos	Pos	Pos	Pos	Pos
188	Rondonia	BRP 49	1800	0	Pos	Pos	Pos	Pos	Pos	Pos	Pos	Pos
189	Rondonia	BRP 50	1800	0	Pos	Pos	Pos	Pos	Pos	Neg	Neg	Pos
190	Rondonia	BRP 51	1800	0	Pos	Pos	Pos	Pos	Pos	Pos	Pos	Pos
191	Rondonia	BRP 52	1200	0	Pos	Pos	Pos	Pos	Pos	Pos	Pos	Pos
192	Rondonia	BRP 53	300	0	Pos	Pos	Pos	Pos	Pos	Pos	Pos	Pos
193	Rondonia	BRP 54	30	0	Pos	Pos	Pos	Pos	Pos	Pos	Pos	Pos
194	Rondonia	BRP 55	90	0	Pos	Pos	Neg	Pos	Pos	Pos	Neg	Pos
195	Rondonia	BRP 56	120	0	Pos	Pos	Pos	Pos	Pos	Pos	Pos	Pos
196	Rondonia	BRP 57	240	0	Pos	Pos	Pos	Pos	Pos	Pos	Pos	Pos
197	Rondonia	BRP 58	60	0	Pos	Pos	Pos	Pos	Pos	Pos	Pos	Pos
198	Rondonia	BRP 59	1800	0	Pos	Pos	Pos	Pos	Pos	Pos	Pos	Pos
199	Rondonia	BRP 60	90	0	Pos	Pos	Pos	Pos	Pos	Pos	Neg	Pos
200	Rondonia	BRP 61	120	0	Pos	Pos	Pos	Pos	Pos	Pos	Pos	Pos
201	Rondonia	BRP 62	120	0	Pos	Pos	Pos	Pos	Pos	Pos	Pos	Pos

	M
1	
2	
3	ELISA Data (ng/mL)
4	not detectable
5	not detectable
6	not detectable
7	163.2
8	20.6
9	not detectable
10	0.7
11	not detectable
12	not detectable
13	40.9
14	1.5
15	100.9
16	not detectable
17	not detectable
18	60.2
19	117.7
20	28.7
21	not detectable
22	14.4
23	not detectable
24	36.8
25	not detectable
26	not detectable
27	not detectable
28	158.6
29	not detectable
30	19.5
31	5.4
32	1.4
33	343.9
34	220.0
35	240.2
36	not detectable
37	not detectable
38	2.3
39	32.3
40	0.7
41	24.9
42	5.5
43	14.8
44	16.4
45	not detectable
46	5.9
47	0.1
48	0.1
49	0.6
50	1.5
51	3.3
52	not detectable
53	7.4
54	not detectable
55	34.4

	M
56	20.0
57	not detectable
58	0.1
59	0.8
60	not detectable
61	not detectable
62	5.8
63	0.0
64	4.3
65	not detectable
66	0.9
67	not detectable
68	not detectable
69	0.7
70	2.4
71	not detectable
72	not detectable
73	not detectable
74	not detectable
75	not detectable
76	46.2
77	6.1
78	not detectable
79	not detectable
80	not detectable
81	not detectable
82	14.7
83	13.8
84	62.1
85	24.8
86	not detectable
87	not detectable
88	not detectable
89	not detectable
90	not detectable
91	32.8
92	not detectable
93	not detectable
94	not detectable
95	not detectable
96	1.0
97	not detectable
98	not detectable
99	not detectable
100	not detectable
101	373.9
102	39.6
103	not detectable
104	not detectable
105	24.7
106	not detectable
107	not detectable
108	16.1
109	86.8
110	0.9
111	0.5
112	37.1

	M
113	252.3
114	107.9
115	4.4
116	107.6
117	0.5
118	0.2
119	not detectable
120	2.3
121	5.6
122	21.5
123	26.5
124	90.8
125	93.4
126	0.1
127	0.3
128	16.2
129	0.4
130	2.5
131	87.7
132	118.8
133	1.5
134	86.4
135	67.8
136	0.3
137	17.3
138	0.9
139	57.8
140	454.0
141	36.3
142	120.8
143	0.8
144	10.1
145	151.7
146	101.6
147	22.2
148	109.3
149	47.7
150	303.5
151	25.8
152	not detectable
153	78.8
154	353.8
155	674.1
156	40.6
157	18.1
158	13.7
159	not detectable
160	20.2
161	108.0
162	338.2
163	162.7
164	7.3
165	159.8
166	3.6
167	1.8
168	not detectable
169	407.9

	M
170	216.0
171	0.7
172	3.9
173	not detectable
174	5.7
175	not detectable
176	119.5
177	189.6
178	40.5
179	not detectable
180	not detectable
181	7.9
182	3.6
183	not detectable
184	6.0
185	4.4
186	1.3
187	15.6
188	7.9
189	4.3
190	153.3
191	182.2
192	266.1
193	3.0
194	not detectable
195	12.7
196	117.5
197	not detectable
198	118.7
199	0.4
200	not detectable
201	not detectable
